# FOXO1-mediated autophagy regulation by miR-223 in sepsis-induced immunosuppression

**DOI:** 10.3389/fphar.2024.1469286

**Published:** 2024-10-08

**Authors:** Guoan Xiang, Qi Li, Di Lian, Chengcheng Su, Xin Li, Shoulong Deng, Lixin Xie

**Affiliations:** ^1^ College of Pulmonary and Critical Care Medicine, Chinese PLA General Hospital, Beijing, China; ^2^ Chinese PLA Medical School, Beijing, China; ^3^ Department of Tuberculosis, Beijing Chest Hospital, Capital Medical University, Beijing, China; ^4^ Department of Respiratory and Critical Care Medicine, Pingjin Hospital, Characteristic Medical Center of the Chinese People’s Armed Police Force, Tianjin, China; ^5^ Department of Emergency, Third Medical Center of Chinese PLA General Hospital, Beijing, China; ^6^ National Center of Technology Innovation for Animal Model, National Human Diseases Animal Model Resource Center, National Health Commission of China (NHC) Key Laboratory of Comparative Medicine, Institute of Laboratory Animal Sciences, Chinese Academy of Medical Sciences and Comparative Medicine Center, Peking Union Medical College, Beijing, China

**Keywords:** sepsis, miR-223, CD4^+^ T lymphocytes, autophagy, FOXO1, immunosuppression

## Abstract

**Introduction:**

Immunosuppression is the main cause of the high mortality rate in patients with sepsis. The decrease in the number and dysfunction of CD4^+^ T lymphocytes is crucial to the immunosuppressed state of sepsis, in turn affecting the development and prognosis of sepsis. Autophagy has been shown to play an important role in the immune imbalance exhibited during sepsis.

**Methods:**

In this study, we modulate the expression of miR-223 in CD4^+^ T lymphocytes, via the transfection of a mimic or an inhibitor of miR-223 to establish cell models of miR-223 overexpression and knockdown, respectively. Levels of autophagy were monitored using a double-labeled lentivirus (mRFP-GFP-LC3) and electron microscopy, and western blot analysis was used to estimate the levels of autophagy-related proteins and FOXO1 in the two cell models after co-treatment with lipopolysaccharide (LPS) and siRNA against FOXO1.

**Results:**

We found that when the expression of miR-223 increased, FOXO1 expression decreased and autophagy decreased; whereas, when FOXO1 expression was inhibited, autophagy decreased significantly in different cell models after LPS induction.

**Conclusion:**

Thus, this study proved that miR-223 participate in the regulation of LPS-induced autophagy via the regulation of FOXO1 expression in CD4^+^ T lymphocytes which shed a new light for the diagnosis and treatment of sepsis.

## 1 Introduction

Sepsis refers to severe organ dysfunction due to an infection (Seymour et al., 2016). It has an acute onset and leads to a high mortality rate; it is a particularly difficult problem in the field of critical respiratory illnesses. In recent years, the incidence of sepsis is on the rise, and the lack of an effective treatment has become one of the leading causes of human death, as sepsis is one of the leading factors contributing to the immediate death of hospitalized patients (Stevenson et al., 2014; Walkey et al., 2015). Thus, there is an urgent need to find effective solutions for the treatment of sepsis (Hotchkiss et al., 2013b; Franks et al., 2015; Shankar-Hari et al., 2015). Previous studies have shown that an immune imbalance plays a key role in the development of sepsis. During immune dysfunction, immunosuppression, and immune paralysis, the innate and adaptive immune systems cannot eliminate pathogens and harmful substances from the body and any additional stressors can lead to aggravation of the existing condition and even death of the patient. T lymphocyte dysfunction is the most important aspect of immune dysfunction during sepsis; however, the underlying mechanism remains unknown (Stieglitz et al., 2015; Bermejo-Martin et al., 2016; Delano and Ward, 2016). Decreased numbers, reduced function, exhaustion, and apoptotic clearance of T lymphocytes are directly related to the poor prognosis of sepsis, and these findings are consistent with our previous study, wherein septic patients exhibited a lower proportion of CD4^+^ T lymphocytes than that in healthy patients. Thus, health of patients with sepsis may worsen, develop multiple organ dysfunction, and even die (Hotchkiss et al., 2013a; Boomer et al., 2014; Hotchkiss and Sherwood, 2015; Luan et al., 2015). CD4^+^ T lymphocytes act as early biomarkers of sepsis and impact the prognosis (Li J. et al., 2015). Further, autophagy of T lymphocytes leads to the expression of abnormal or aged proteins and organelles and excessive peroxide-mediated degradation of proteins. Thus, programmed cell death during immunosuppression might be the key to solving the immune imbalance exhibited during sepsis.

In patients with HIV, nearly 10 micro RNAs (miRs) were found to regulate CD4^+^ T lymphocyte numbers and affect the replication and infection ability of the virus. In a previous study, we used Solexa sequencing to analyze circulating microRNAs in the early stages of sepsis to determine whether there were any microRNAs that functioned as biomarkers of sepsis. Thus, we identified microRNAs differentially expressed between septic and healthy patients, and among them, miR-223, which is known to have a profound impact on the differentiation of blood cells and on granulocytes and T lymphocytes, was found to exhibit higher sensitivity and specificity in predicting the prognosis of septic patients (Wang et al., 2012; Wang et al., 2014). High expression of miR-223 has also been detected in the peripheral blood and synovial T lymphocytes of patients with rheumatoid arthritis, and miR-223 possibly impacts the development of arthritis and inflammatory bowel disease (IBD) by affecting the activity of immune cells (Fasseu et al., 2010). Although many studies have found that some miRNAs regulate autophagy levels in cells and in turn affect the prognosis in animal disease models, its role in regulating autophagy levels of immune cells in patients of sepsis is still unknown.

In our previous study, we used TargetScan and miRanda, in conjunction with the Kyoto Encyclopedia of Genes and Genomes pathway and the gene ontology enrichment analysis, and predicted the gene encoding the forkhead box protein O1 (FOXO1) as one of the target genes of miR-223, and studies have also shown that FOXO1, as a miR-223 downstream target, plays a role in cancer development (Haneklaus et al., 2013). As a member of the FOX transcription gene family, subtype O, FOXO1 is widely expressed in eukaryotes and is located on human chromosome 13q14.1. FOXO1 is also known to participate in the PI3K/AKT pathway, and its downstream targets are involved in many important cellular biological processes, such as apoptosis (Fu and Tindall, 2008), cell cycle inhibition, oxidative stress tolerance, anabolism, and catabolism (Du et al., 2020).

In this study, we modulated miR-223 expression in CD4^+^ T lymphocytes using chemically synthesized miRs and estimated the autophagy levels after lipopolysaccharide (LPS) treatment. We also inhibited the expression of FOXO1 using small interfering (si) RNA technology, and subsequently detected changes in the level of autophagy after LPS treatment, in order to explore the possible pathways by which miR-223 regulates the levels of autophagy during sepsis and find a potential treatment for sepsis.

## 2 Materials and methods

### 2.1 Reagents and materials

CD4 (L3T4) MicroBeads, LS columns with pre-separation filters (70 µm) were purchased from Miltenyi Biotec, United States. Two milliliters of CD4 (L3T4) MicroBeads were conjugated to monoclonal anti-mouse CD4 antibody (L3T4; isotype: rat IgG2b). IFN gamma Mono-clonal Antibody (XMG1.2), APC, eBioscience™ was purchased from Thermo Fisher, and Anti-IL-4 antibody [11B11] (PE) (ab93503), recombinant Anti-Annexin V/ANXA5 anti-body [EPR3980] (ab108194) purchased from Abcam. The miR-223 mimic (chemically synthesized double-stranded mature miRNA), and miR-223 inhibitor (Chemically synthesized mature miRNA complementary single-stranded) were purchased from Biomics Biotechnology Co., Ltd. The study design was approved by the appropriate ethics review board. Synthesized microRNAs were purchased from Biomics, China. siRNA-FOXO1 was purchased from Shenggong Bioengineering Co., Ltd., China. The primer sequences used in this experiment are including miR-223 (F: 5′-ACA​CTC​CAG​CTG​GGA​CCC​CAT​AAA​CTG​TTT-3′, R: 5′-TGG​TGT​CGT​GGA​GTC​G-3′), U6 (F: 5′-CTC​GCT​TCG​GCA​GCA​CA-3′, R: 5′-AAC​GCT​TCA​CGA​ATT​TGC​GT-3′), FOXO1 (F: 5′-GAG​CGT​GCC​CTA​CTT​CAA-3′, R: 5′-CCA​TCT​CCC​AGG​TCA​TCC-3′), P62(F: 5′-CAG​ATG​CCA​GAA​TCG​GAA​G-3′, R: 5′-GGT​CTG​TAG​GAG​CCT​GGT​GAG-3′), LC3B (F: 5′-CCC​CAC​CAA​GAT​CCC​AGT-3′, R: 5′- CGC​TCA​TGT​TCA​CGT​GGT-3), β-actin (F: 5′- AAG​GAG​CCC​CAC​GAG​AAA​AAT-3′, R: 5′- ACC​GAA​CTT​GCA​TTG​ATT​CCA​G-3′). The miRcute miRNA extraction and separation kit (spin column type) and the miRcute enhanced miRNA cDNA first strand synthesis kit were purchased from Tiangen Bio-chemical Technology Co., Ltd., China. Lipofectamine 2000 was purchased from Thermo Fisher, United States, and LPS was purchased from Sigma-Aldrich, United States. Double-labeled lentivirus (mRFP-GFP-LC3) was purchased from Hanbio Biotechnology Co., Ltd., China. CD3 antibody and CD28 antibody were purchased from Thermo Fisher, United States.

### 2.2 Mice housing and sample tissue collection

Specific pathogen-free healthy 10-weeks old C57BL/6J male or female mice SPF grade, weighing about 20 g, were purchased from the Sbefu (Beijing) Biotechnology Co., Ltd. (license number: SCXK-(jing) 2019-0010). The experiment comply with the ARRIVE guidelines and be carried out in accordance with the National Institutes of Health guide for the care and use of Laboratory animals (NIH Publications No. 8023, revised 1978). We obtained ethical approval from the Research Ethics Committee of the Institute of Microbiology, Chinese Academy of Sciences, with the approval number APIMCAS2022143. The mice were euthanized by cervical dislocation, and subsequently, spleen tissues were collected for the isolation of CD4^+^ T lymphocytes.

### 2.3 Isolation of CD4^+^ T lymphocytes and TCR activation

Magnetic bead sorting was used to sort CD4^+^ T lymphocytes from the spleen of C57BL/6J mice, and flow cytometry was used to detect the proportion of CD4^+^ T lymphocytes and the number of viable cells. The whole process was performed under sterile conditions. The cells were put into the well plate coated with CD3e and CD28 antibody for 72 h, and CD4^+^ T lymphocytes were cultured at 5% CO2 and 37°C. Roswell Park Memorial Institute (RPMI) 1640 medium with 10% fetal bovine serum (FBS), density, and the number of cells were estimated using a Nikon E600POL polarized light microscope.

### 2.4 Transfection of CD4^+^ T lymphocytes

To manipulate miR-223 expression levels in CD4^+^ T lymphocytes, we employed synthetic miR-223 mimics and inhibitors, following the product instructions and adhering to the recommended dosage concentrations. Specifically, the experimental groups were divided into two cohorts: one subjected to the miR-223 mimic treatment, which involved the transfection of a 50 nM final concentration of miR-223-5p mimic to augment miR-223 levels; and the other to the miR-223 inhibitor treatment, characterized by the administration of a 100 nM final concentration of miR-223-5p inhibitor to attenuate miR-223 expression. Parallel control groups were established for both the miR-223 mimic and inhibitor treatments. The success of transfection was estimated using real-time polymerase chain reaction to quantify miR-223 expression in each group 48 h post-transfection. Subsequently, the cells were treated with 20 nM Bafilomycin A1 (BafA1) for 4 h, centrifuged at 1,000 rpm for 5 min at room temperature, followed by the addition of RPMI 1640, containing 10% fetal bovine serum (FBS), and incubation with 1 μg/mL LPS for 18 h.

### 2.5 Estimation of autophagy levels and differentiation in LPS-treated CD4^+^ T lymphocytes

Observation of the number of autophagosomes in different groups of cells under electron microscopy. Double-labeled lentivirus (mRFP-GFP-LC3) and Nikon ECLIPSE 80i fluorescence microscope and image acquisition and analysis system were used to estimate the changes in autophagy levels in LPS-treated CD4^+^ T lymphocytes. Fluorescence microscopy were used to observe the green fluorescent protein (GFP), red fluorescent protein (RFP), and estimate the fluorescence intensity at × 400. Antibodies against IFN- γ and IL-4 markers were used to detect the T helper type 1 (Th1) and T helper type 2 (Th2) per-centage in the LPS-treated cells using flow cytometry.

### 2.6 siRNA-FOXO1 transfection

Cells transfected with synthetic miRs and siRNA-FOXO1. After 48 h, the cells were incubated with 20 nM BafA1 for 4 h, centrifuged at 1,000 rpm for 5 min at room temperature, followed by addition of RPMI 1640 medium, containing 10% FBS, and incubation with 1 μg/mL LPS for 18 h.

### 2.7 Western-blotting

The western blotting technique was used to detect the differences in the protein expression levels of FOXO1, P62, IL3B between the cells in each group treated with LPS 1 ug/ml for 18 h. In brief, total protein was extracted from each group using a RIPA lysis buffer. Protein concentration was then detected using a BCA (bicinchoninic acid) protein assay kit. SDS-PAGE was conducted, and the protein was electrotransferred to a polyvinylidene fluoride (PVDF) membrane. It was blocked with 5% skim milk and incubated for 2 h at room temperature, followed by overnight incubation at 4°C with primary antibody. After washing with TBST, HRP-labeled protein bands were visualized using an ECL kit. The bands were analyzed using ImageJ software with β-actin as an internal reference.

### 2.8 Statistical analysis

A two-tailed Student’s t-test or a one-way analysis of variance (ANOVA) was per-formed when comparing two groups or more than two groups, respectively. The normality test was checked by the Shapiro–Wilk test and equal variance was checked by Levene’s test. Statistical analysis was performed using Microsoft Excel LTSC 2021 and Prism 9.0 (GraphPad). Data are expressed as means ± SEM. Difference was considered to be significant if *p* < 0.05 (*p < 0.05, **p < 0.01, ***p < 0.001, unless otherwise indicated).

## 3 Results

### 3.1 CD4^+^ T lymphocytes capture

**FIGURE 1:**
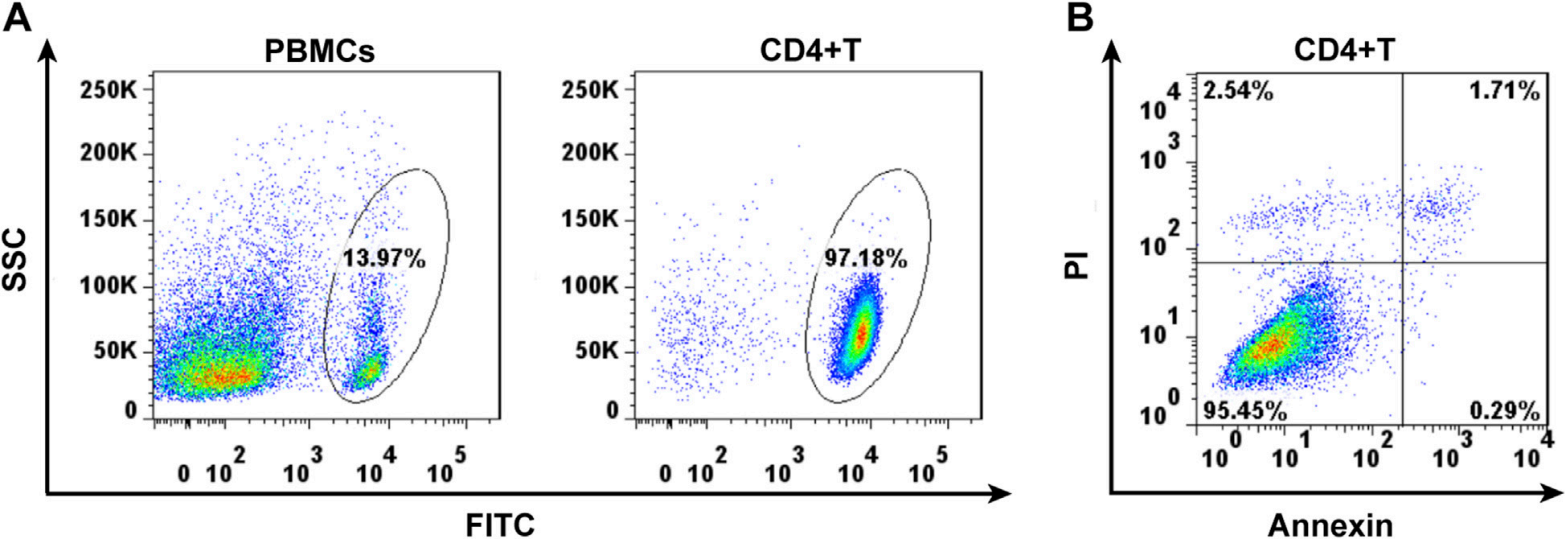
Cell separation of the CD4^+^ lymphocytes by magnetic cell sorting **(A)** and cell viability detected by Annexin using flow cytometry **(B)**.

The surviving CD4^+^ T lymphocytes were screened from mouse spleen cell suspension by magnetic bead sorting technique (Figure 1A). Annexin revealed that 95% of CD4^+^ T cells, sorted using flow cytometry, were living (Figure 1B).

### 3.2 miR-223 affects the health of LPS-treated CD4^+^ T lymphocytes

**FIGURE 2:**
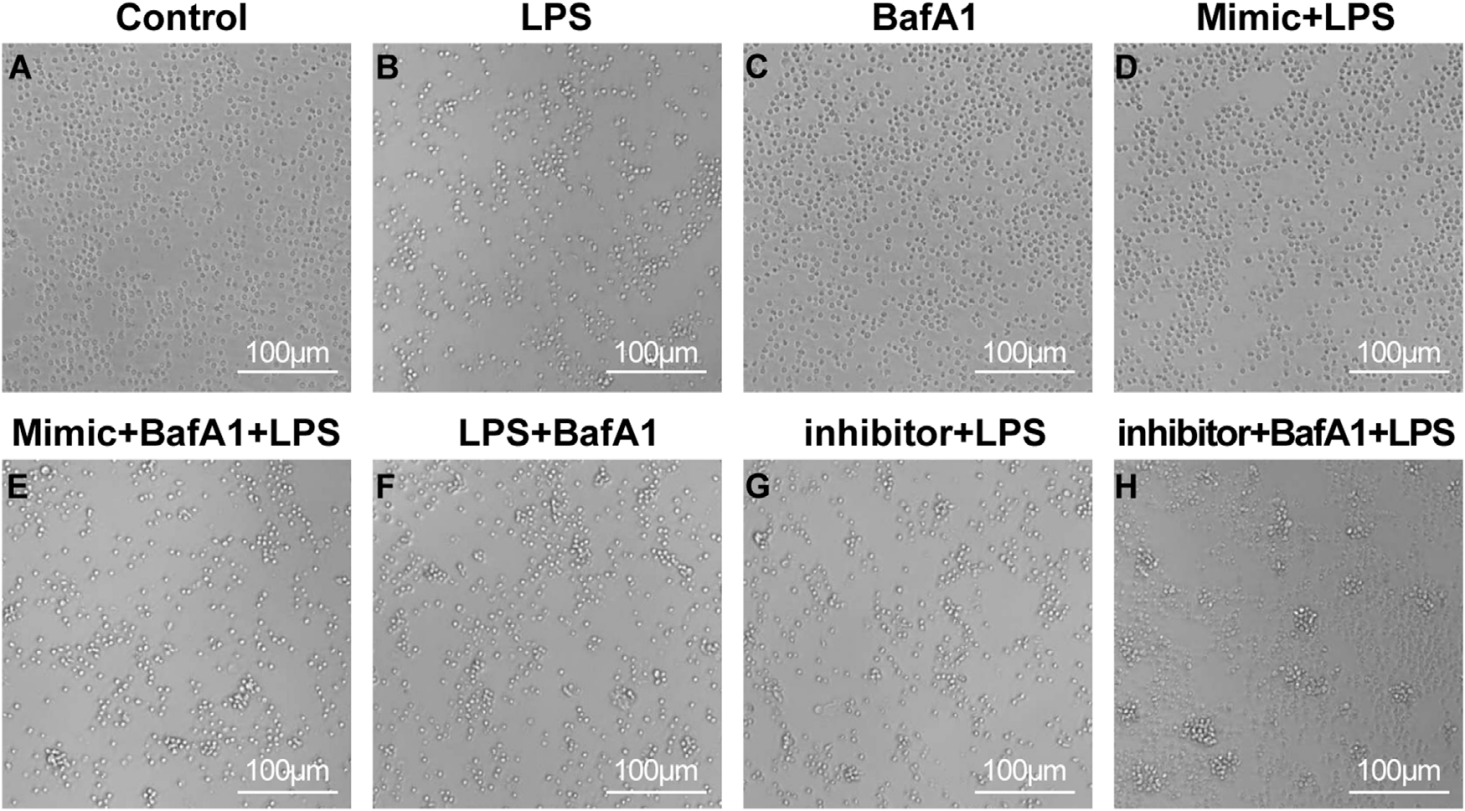
The survival state of CD4 + T lymphocytes after various interventions under the micro-scope. **(A–H)** The cell morphology was control group, LPS group, BafA1 group, mimic + LPS group, mimic + BafA1 + LPS group, LPS + BafA1 group, inhibitor + LPS group and inhibitor + BafA1 + LPS group, respectively.

The sorted CD4^+^ T cells were cultured for 24 h and given different interventions respectively. The cells of different groups could be observed to present different cell states through microscopy (Figure 2). In the control group, the cell count filled the entire field of view, with cells exhibiting a plump morphology and no evidence of cell clumping. In the control group, the cell count filled the entire field of view, with cells exhibiting a plump morphology and no evidence of clumping cells. The cells in the BafA1 group showed a similar state to the control. In contrast, in the remaining groups, under LPS stimulation, cells exhibited aggregation and a significant reduction in number. However, the miR-223 mimic corrected the effects induced by LPS, with the cell state resembling that of the control group, while the miR-223 inhibitor exacerbated the damage to the cells caused by LPS.

### 3.3 miR-223 affects the autophagosome formation in LPS-treated CD4^+^ T lymphocytes

**FIGURE 3:**
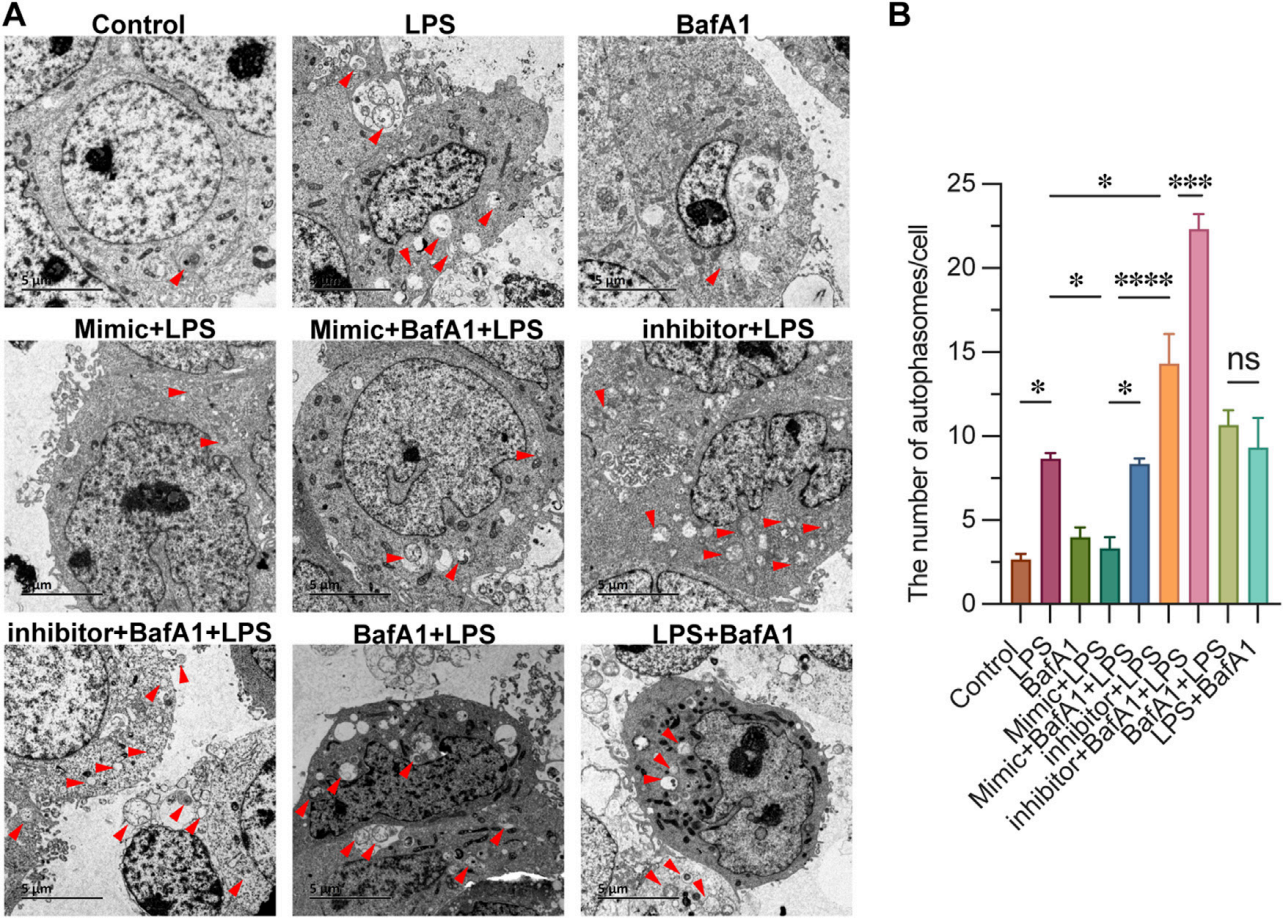
miR-223 affects the autophagosome formation in LPS-treated CD4^+^ T lymphocytes. **(A)** Representative images of autophagosomes, observed under an electron microscope, in cells of each group (red arrow). Scale bars: 5 µm. **(B)** The quantitation data were represented with 3 replicates. *p < 0.05, ***p < 0.001, ****p < 0.0001. error bars represent mean ± SEM.

To investigate the regulatory role of miR-223 on autophagy activity, we employed electron microscopy to observe and assess the levels of autophagy in each group, and quantified the number of autophagosomes within individual cells. As shown in Figure 3, The typical phenotype of CD4^+^ T lymphocytes, characterized by a large nucleus and rare autophagosome structures, was observed in the Control group. In the LPS group, we observed abundant accumulation of autophagosomes in CD4^+^ T lymphocytes, confirming the activation of autophagy. This accumulation was also evident in both the BafA1 + LPS group (where BafA1 was treated first) and the LPS + BafA1 group (where LPS was treated first), regardless of the order of BafA1 treatment. Following treatment with the miR-223 mimic in mimic + LPS group, the absence of autophagosome accumulation suggested an impairment of autophagy. Conversely, the inhibitor + LPS group showed the opposite effect. Here, we document that the miR-223 mimic inhibits LPS-induced autophagosome formation in CD4^+^ T lymphocytes.

### 3.4 Cell transfected with the miR-223 mimic showed lower levels of autophagy

**FIGURE 4:**
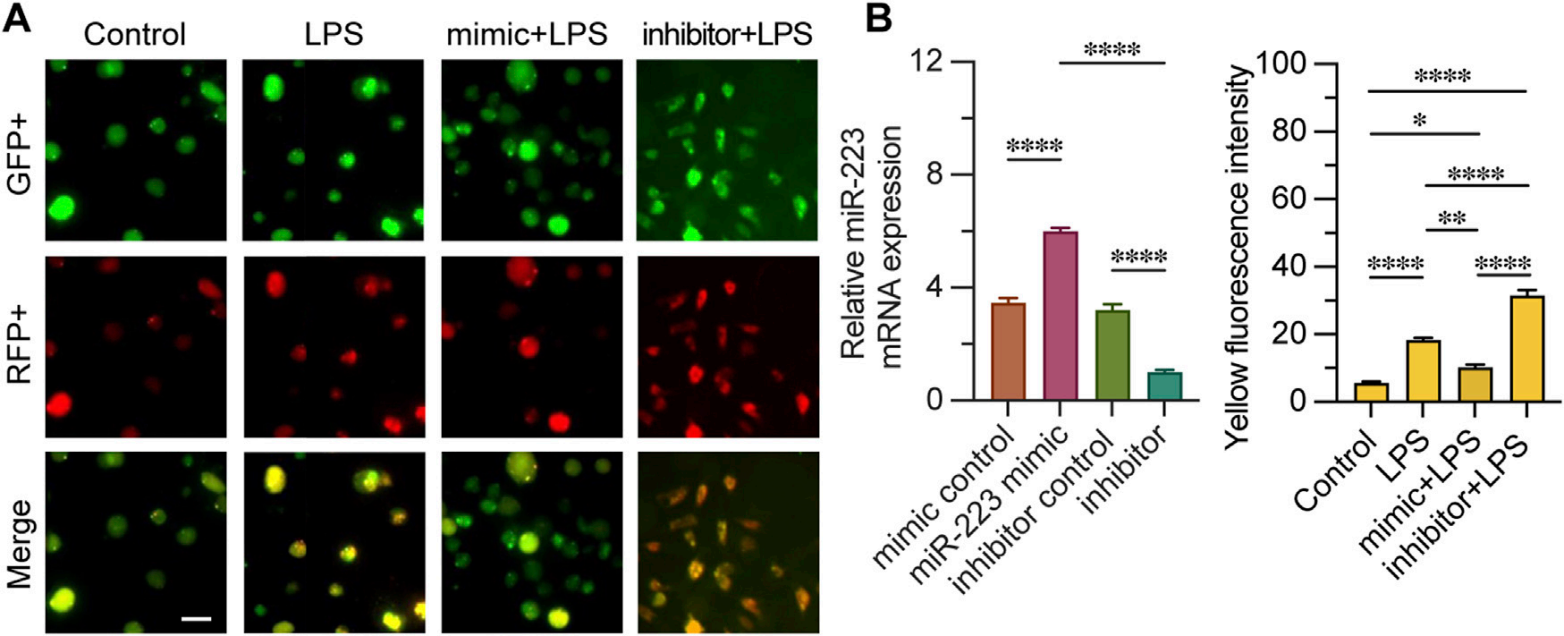
Quantitative analysis of mRFP-GFP-LC3 fluorescence monitoring autophagy process fluorescence intensity. **(A)** observation of mRFP-GFP-LC3 autophagy by fluorescence microscope in groups mimic + LPS, inhibitor + LPS, LPS and control. **(B)** Relative miR-223 mRNA expression was examined to verify whether transfection was effective, the fluorescence intensity of both GFP+ and RFP+ was measured in each group; n = 3. Scale bar represents 10 μm. *, **p < 0.05, ***p < 0.001, ****p < 0.0001, ns *p* > 0.05.

We evaluated the impact of miR-223 mimic or inhibitor on autophagy by transfecting CD4^+^ T lymphocytes with an mRFP-GFP-LC3 expression construct. To confirm the efficacy of transfection, we quantified the relative expression levels of miR-223 mRNA. Fluorescence microscopy was utilized to assess the fluorescence intensity of cells exhibiting mRFP-GFP-LC3 punctae across different groups. The merging of GFP and RFP fluorescence channels revealed that orange-red punctae are indicative of active autophagy within the cells (Figure 4). Upon LPS stimulation, an increased fluorescence intensity of mRFP-GFP-LC3 punctae was observed compared to the control group, suggesting that LPS triggers autophagy. Notably, the miR-223 mimic treatment significantly diminished the intensity of the orange-red fluorescence in cells with mRFP-GFP-LC3 punctae. Conversely, the inhibition of miR-223 exacerbated the effects of LPS, as evidenced by the increased accumulation of orange-red punctae. Collectively, these findings indicate that the miR-223 mimic inhibits LPS-induced autophagy in CD4^+^ T lymphocytes.

### 3.5 miR-223 affects Th1/Th2 differentiation in LPS-treated CD4^+^ T lymphocytes

**FIGURE 5:**
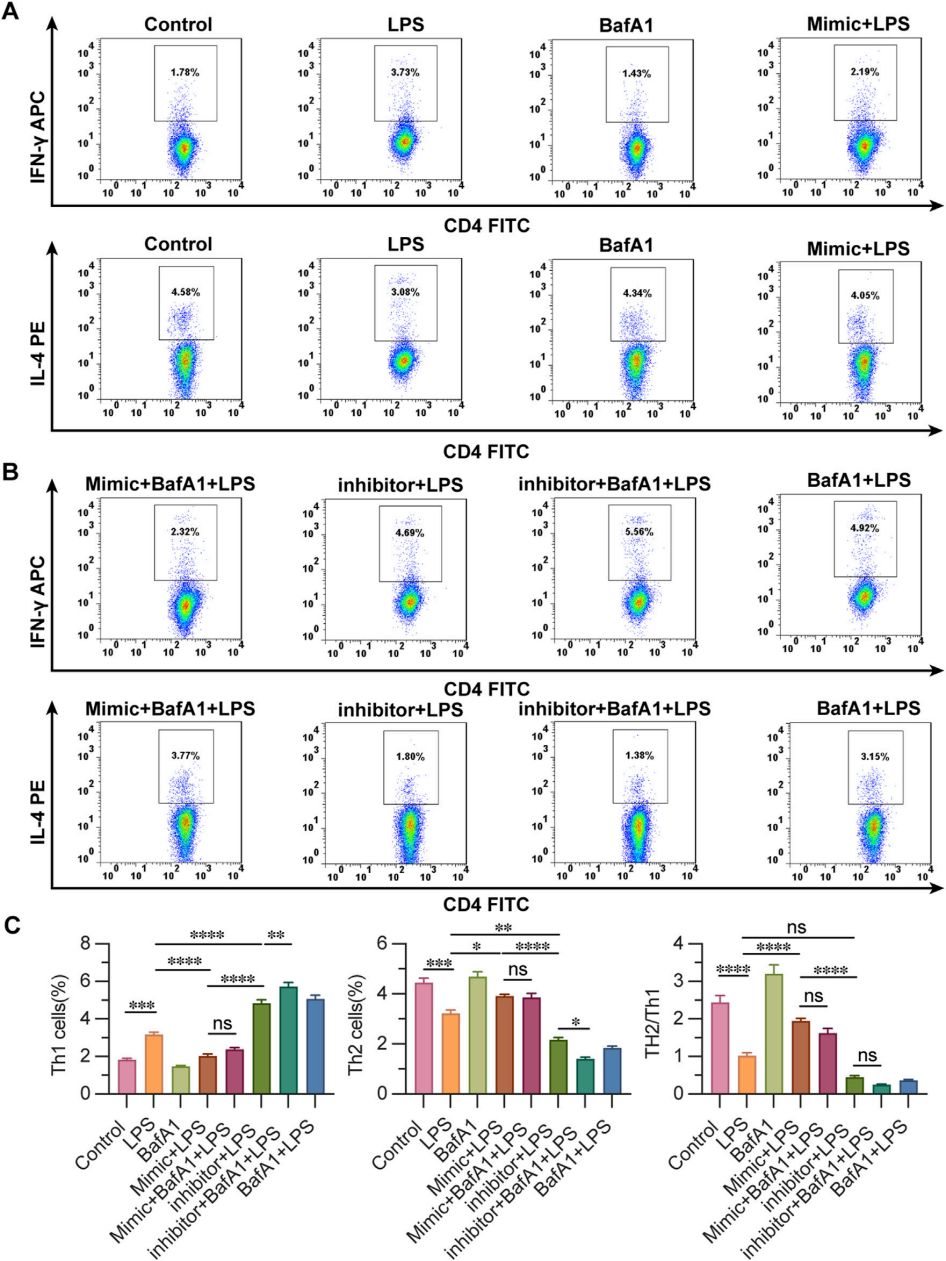
Detection of the ratio of Th2/Th1 differentiation of CD4^+^ T lymphocytes using flow cytometry. **(A, B)** observation of the ratio of Th2/Th1 differentiation of CD4^+^ T lymphocytes by flow cytometry in groups. **(C)** The ratio of Th1 cells and Th2 cells by flow cytometry in groups. Results are represented as mean ± SEM, n = 3. *, **p < 0.05, ***p < 0.001, ****p < 0.0001, ns *p* > 0.05.

IFN- γ, IL-4 labeled the Th1 and Th2 ratio of cells in each group, evaluating the effect of miR-223 on the Th2/Th1 differentiation of CD4+ T cells in each group. The percentage of Th1 cells labeled with IFN-γ showed that the proportion of control group and BafA1 group was the lowest, followed by mimic + LPS group and mimic + BafA1 + LPS group, and the ratio of Th1 cells was the highest in inhibitor + BafA1 + LPS group, followed by BafA1 + LPS group and inhibitor + LPS group. The results showed that the proportion of Th2 cells in BafA1 group and control group were the highest, followed by mimic + LPS and mimic + BafA1 + LPS group, and the ratio of Th2 cells in inhibitor + BafA1 + LPS group was the lowest, followed by the group of BafA1 + LPS, inhibitor + LPS and LPS. It is considered that the increased miR-223-expression makes CD4 + T lymphocytes more likely to differentiate into Th2 cells. It is suggested that the change of miR-223 expression affects the Th2/Th1 differentiation ratio of LPS-affected CD4 + T lymphocytes (Figure 5).

### 3.6 miR-223 affects mRNA levels of FOXO1 and P62

**FIGURE 6:**
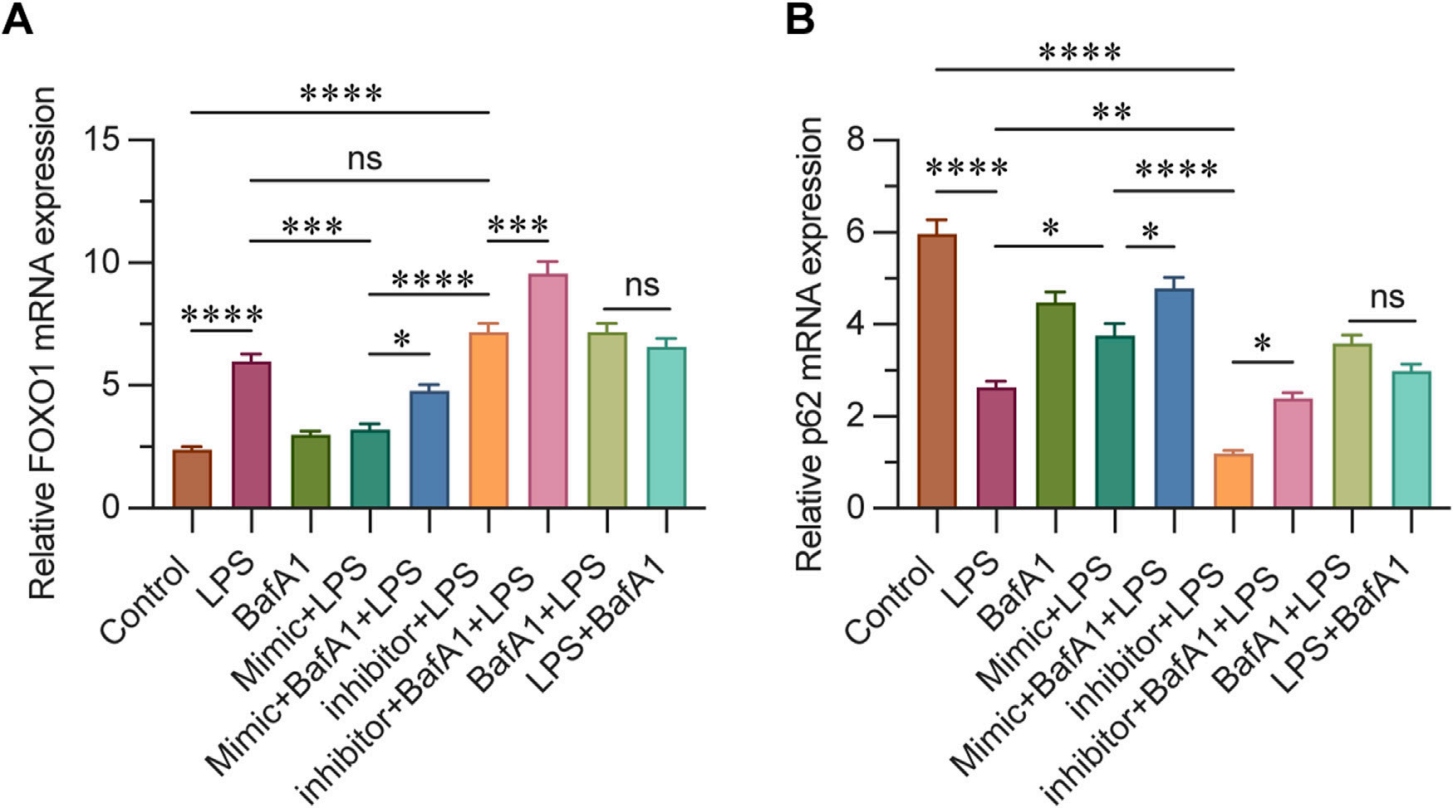
**(A)** Relative mRNA levels of FOXO1 in each group were determined using qPCR. **(B)** Relative mRNA levels of P62 in each group were determined using qPCR. β-actin was used as the loading control. Results are represented as mean ± SEM, n = 3. *, **p < 0.05, **p < 0.01, ***p < 0.001, ****p < 0.0001, ns *p* > 0.05.

The mRNA expression levels of FOXO1 and p62 were assessed using real-time quantitative PCR, and the activation level of autophagy was evaluated at the mRNA level. p62 is a crucial autophagy substrate, and its expression level can directly reflect the intensity of autophagy activity. In this study, the results indicated that the expression of FOXO1 in the control and BafA1 groups was significantly lower compared to other groups, with the mimic + LPS group showing an intermediate level. Notably, the highest expression of FOXO1 was observed in the inhibitor + BafA1 + LPS group. These findings suggest that FOXO1 expression decreases as miR-223 expression increases (Figure 6A). Conversely, p62 expression was highest in the control group, with the mimic + BafA1 + LPS group exhibiting a higher level than the mimic + LPS group, and the lowest level found in the inhibitor + LPS group. This suggests that following LPS intervention, p62 mRNA expression increases with the upregulation of miR-223 (Figure 6B).

### 3.7 miR-223 regulates the expression of FOXO1 and autophagy-related proteins

**FIGURE 7:**
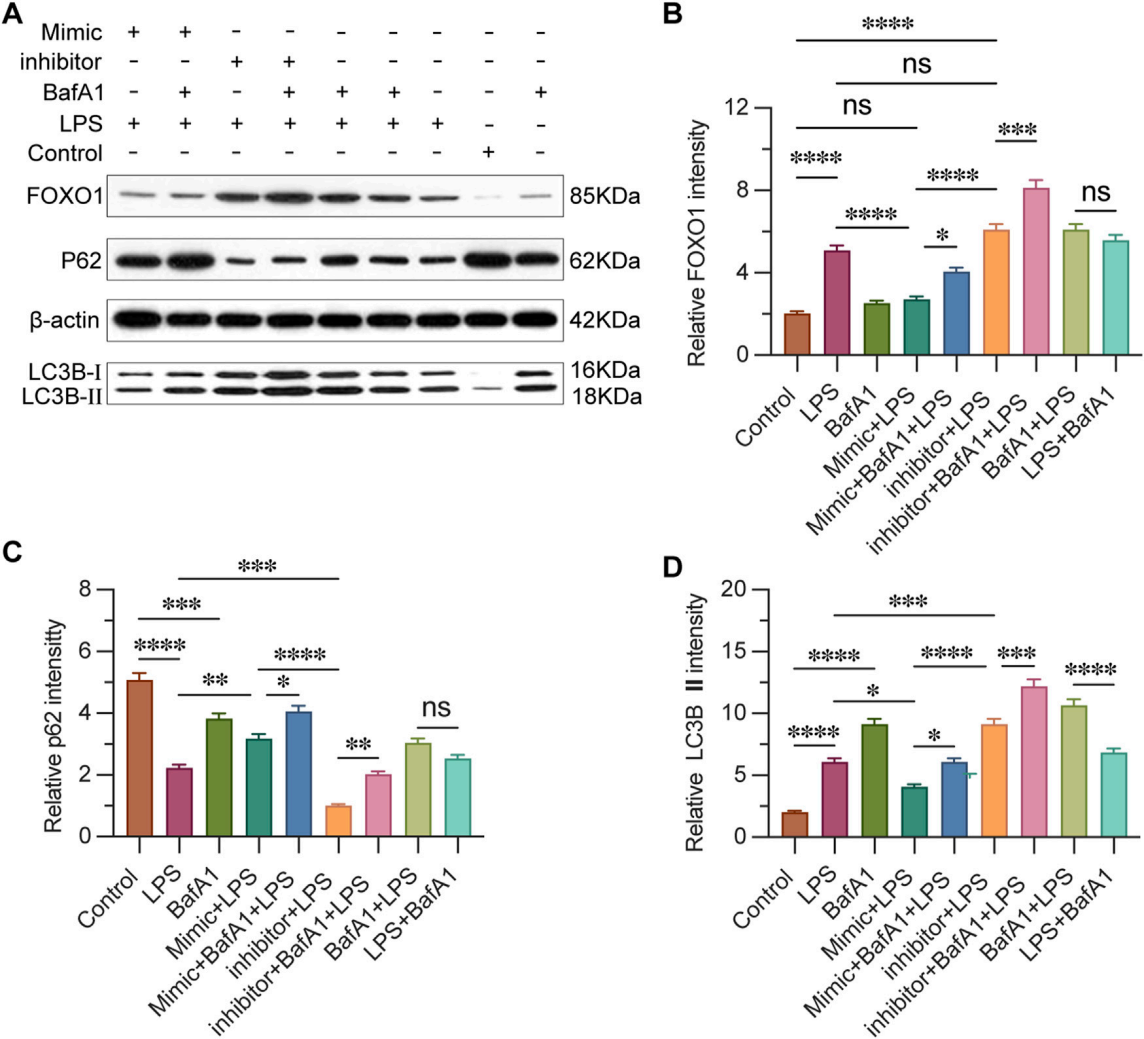
miR-223 affects the expression of FOXO1, autophagy-related proteins. **(A)** Western blot analysis of the expression of FOXO1 and autophagy-related proteins. **(B)** Results are reported as relative intensity values of FOXO1 in each group. **(C)** Results are reported as relative intensity values of p62 in each group. **(D)** Results are reported as relative intensity values of LC3B Ⅱ in each group. β-actin was used as the loading control. Results are represented as mean ± SEM, n = 3. *, **p < 0.05, **p < 0.01, ***p < 0.001, ****p < 0.0001, ns *p* > 0.05.

To evaluate the impact of miR-223 on FOXO1-mediated autophagy in CD4^+^ T lymphocytes, we conducted Western blot analysis to examine the expression levels of FOXO1 and p62, as well as the conversion of LC3, which are proteins associated with autophagy (Figure 7A). The results revealed that the expression level of FOXO1 in the inhibitor + LPS group was significantly higher compared to both the mimic + LPS group and the Control group (Figure 7B). This observation suggests that miR-223 expression indeed regulates FOXO1 expression, which is consistent with previous studies identifying FOXO1 as a target gene of miR-223. In the context of autophagy induction, protein aggregate removal, and autophagy inhibition, p62 labeling serves as a highly informative method. The LC3B label enables the tracking of p62 binding and the subsequent replenishment of autophagosomes. Typically, a decrease in LC3BII and an increase in p62 expression are indicative of autophagy activation (Yoshii and Mizushima, 2017). Our results demonstrated that in the presence of BafA1, LC3-II accumulated under all conditions, including in untreated and LPS-treated cells. Furthermore, we observed that p62 expression was lowest in the inhibitor + LPS group, followed by the inhibitor + BafA1 + LPS group, with the highest levels detected in the control group and the mimic + BafA1 + LPS group (Figures 7C, D). The expression of autophagy-related proteins was influenced by the levels of miR-223, suggesting that increased miR-223 expression leads to weakened autophagy activity, while decreased miR-223 expression enhances autophagy activity.

### 3.8 FOXO1 regulates autophagy in CD4^+^ T lymphocytes

**FIGURE 8:**
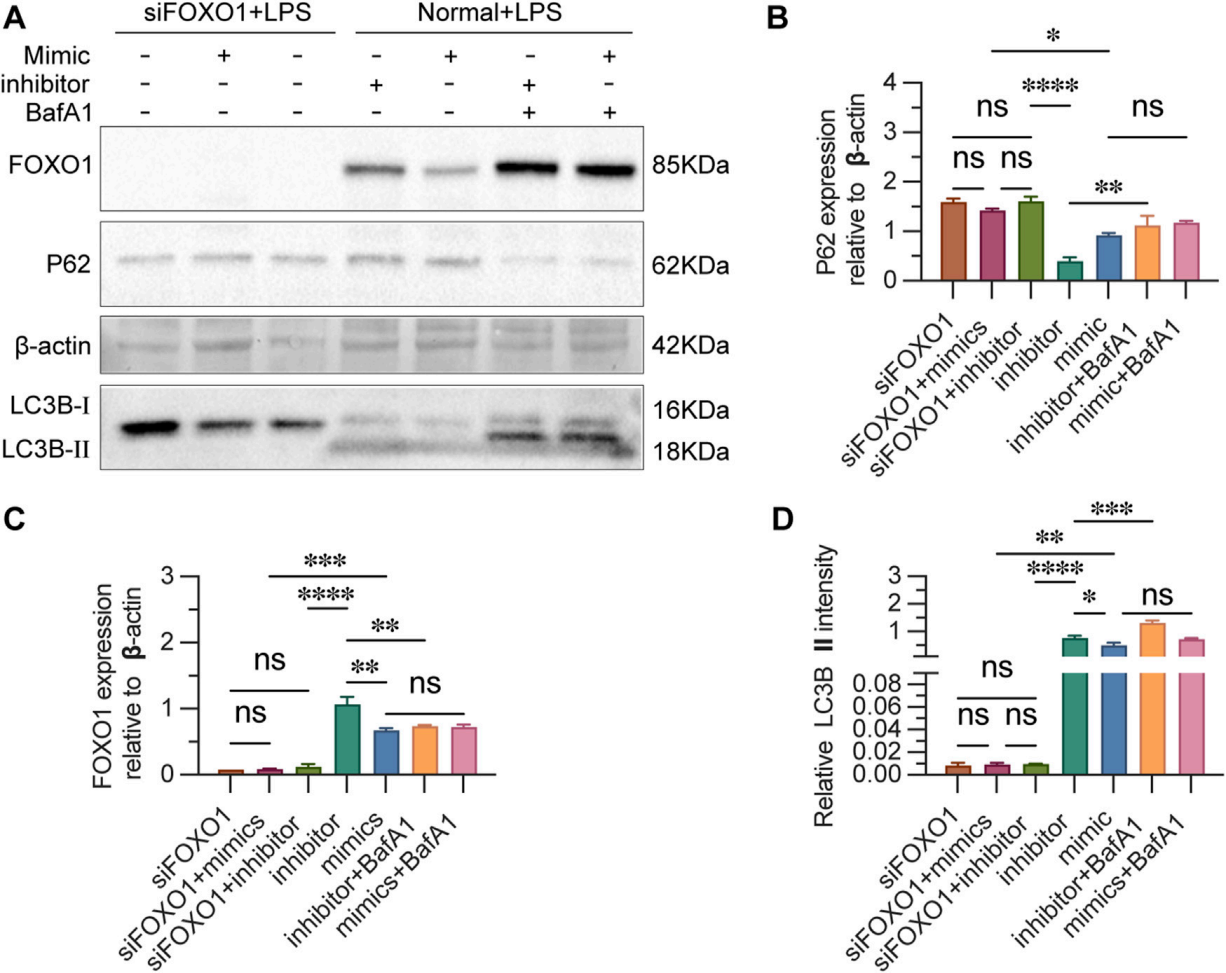
Estimation of FOXO1 and autophagy-related proteins after si-FOXO1 transfection. **(A)** Western blot analysis of the expression of FOXO1 and autophagy-related proteins. **(B)** The results are reported as the relative intensity values of p62in each group. **(C)** The ratios are reported as the intensity values of FOXO1 in each group. **(D)** The results are reported as the relative intensity values of LC3B Ⅱ in each group. β-actin was used as the loading control. Results are represented as mean ± SEM, n = 3. *p < 0.05, **p < 0.01, ***p < 0.001, ****p < 0.0001, ns *p* > 0.05.

To determine whether miR-223 regulates autophagy by regulating FOXO1 in LPS-treated cells. The autophagy activity of cells in each group were detected by autophagy related proteins after blocking the expression of FOXO1. Transfection of cells with siRNA-FOXO1 lead to the death of a large number of cells and the remaining cells were collected to estimate the expression of autophagy-related proteins. Western blot analysis indicated that, in the absence of LPS and inhibitor, the expression levels of FOXO1 and LC3BII proteins were significantly elevated in transfected cells, while the expression of P62 protein was reduced, compared to untransfected cells (Figures 8A–D). The silencing of FOXO1 disrupted the formation of the autophagic flux. Following siRNA-FOXO1 transfection, the expression of P62 protein in the miR-223 mimic group was lower than that in the inhibitor group, although this difference did not reach statistical significance. These findings suggest that FOXO1 is a primary pathway through which miR-223 regulates autophagy in CD4^+^ T lymphocytes.

## 4 Discussion

**FIGURE 9:**
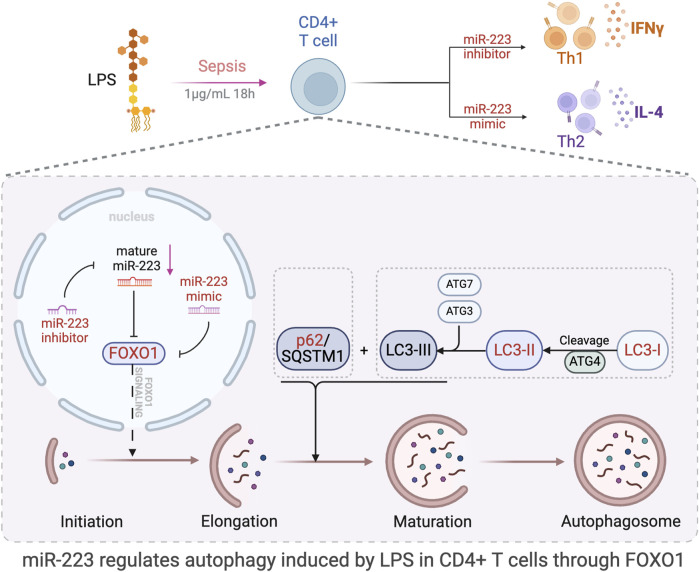
Schematic representation of the study and results. The figure was created in part using BioRender.com. Model of the effect of miR-223 regulation of autophagy through direct targeting of FOXO1. This study proved that miR-223 participate in the regulation of LPS-induced autophagy via the regulation of FOXO1 expression in CD4^+^ T lymphocytes.

The objective of this study was to investigate the role of miR-223 in regulating autophagy and immune function in CD4^+^ T lymphocytes during sepsis, and to elucidate the mechanisms through which miR-223 contributes to the pathophysiology of sepsis. Our goal was to identify potential novel strategies for the diagnosis and treatment of this condition. We discovered a significant correlation between miR-223 and FOXO1, with FOXO1 being a downstream target of miR-223. Specifically, we observed that the expression of FOXO1 decreases with an increase in miR-223 expression, and *vice versa*, at both the protein and mRNA levels. Further, we also demonstrate that with an increase in expression of miR-223, autophagy levels also significantly reduced in CD4^+^ T lymphocytes. When FOXO1 expression was inhibited in CD4^+^ T lymphocytes, autophagy was found to be significantly reduced, indicating that the miR-223-mediated regulation of FOXO1 expression is one of the main pathways through which miR-223 regulates autophagy of CD4^+^ T lymphocytes. We also report that miR-223 mediates the differentiation of CD4^+^ T lymphocytes treated with LPS into Th2 rather than Th1-type cells, indicating that high levels of miR-223 expression plays a role in regulating the Th1/Th2 balance and consequently reducing the incidence of cytokine-mediated hyperimmune responses associated with Th1 in sepsis. The molecular mechanisms by which miR-223 regulates FOXO1-mediated autophagy in LPS-induced sepsis in CD4^+^ T lymphocytes, and its effects on autophagy-related proteins, are summarized in Figure 9.

Sepsis is a life-threatening condition characterized by organ dysfunction and high mortality rates, resulting from the body’s dysregulated response to infection. An exaggerated immune reaction to bacterial pathogens can precipitate sepsis. In this context, sepsis biomarkers play an extremely crucial role in diagnostics, therapeutic monitoring, and prognostic assessment. Our previous work has showed that miR-223 can be used as an early biomarker of sepsis. However, other potential functions and roles of miR-223 in sepsis have not been fully revealed. At the same time, our preliminary literature research found that autophagy is a biological phenomenon whereby components of cells can self-degrade using autophagosomes, the regulation of autophagy defects may potentially be used to treat some diseases (Wang et al., 2023).

The miR-223 has been previously demonstrated to play a role in a variety of biological processes, including development, differentiation, hematopoiesis, and immune system regulation. Recent studies, including those by Li Y. et al., have shed new light on autophagy. They confirmed that in mice, miR-223 deficiency leads to increased ATG16L1 and LC3-II protein expression in bone marrow-derived macrophages. Conversely, ATG16L1 levels were found to decrease in BV2 cells with miR-223 overexpression but increase with antagomir treatment. This suggests that miR-223 regulates autophagy by targeting the 3′UTR of ATG16L1, and that autophagy levels can be restored to normal by overexpressing ATG16L1, even in the presence of miR-223 mimics (Li et al., 2019b). Another study suggests that miR-223-3p expression and related cytokine levels could serve as predictors of response to ECT in individuals with treatment-resistant depressive disorders. They confirm that ROC analysis of confirmed the diagnostic power of miR-223-3p demarcating ECT-responders from non-responder subjects (AUC = 0.76, *p* = 0.0031) (Kaurani et al., 2023). As gene therapy technology advances, the clinical value of miR-223 is expected to become increasingly apparent. Despite the absence of current clinical trials examining the clinical value of miR-223, its therapeutic potential to target inflammatory pathways is emphasized as a means to counteract excessive innate immune responses during mucosal inflammation.

Transgenic miR-223-/Y mice administrated intraperitoneal or intravenous LPS, in order to establish sepsis, exhibited a larger extent of tissue damage compared to that of the controls (Johnnidis et al., 2008). It is interesting to note that miR-223 is expressed at low levels during sepsis. The negative correlation between miR-223 expression and the incidence of sepsis suggests miR-223 downregulation is vital to the development of inflammation and infection in sepsis (Wang et al., 2010). A similar link between miR-223 expression and autophagy levels are seen in patients with atherosclerosis obliterans. As the expression of miR-223 decreases, vascular smooth muscle cells lose the ability to induce cellular autophagy and evolve into foam cells. However, the underlying mechanism of miR-223-mediated regulation of autophagy in CD4^+^ T lymphocytes autophagy and its impact on the prognosis of the disease remains unknown.

LPS is the main pathogenic component of gram-negative bacilli, and it often causes systemic inflammation, blood hypercoagulation, hypothermia, and other fatal complications. Intraperitoneal injection of LPS is a classical method to induce sepsis in mice (Doi et al., 2009; Dejager et al., 2011). The classic pathogenic mechanism of LPS involves the activation of innate immune cells, like macrophages, to secrete inflammatory cytokines through activation of the toll-like receptor 4 (TLR4), including TNF-α, IL-1 β, IL-6, IL-12, and IFN-γ (Li C. C. et al., 2015). Additionally, LPS has pro-inflammatory effects, triggering pulmonary inflammatory responses through nitrogen oxide-dependent redox signaling in pulmonary endothelial cells (Menden et al., 2013), perturbing airway function, and pulmonary circulation in mice (Ni et al., 2012; Lin et al., 2015). Thus, LPS is widely used to induce systemic inflammation in animal models. Of note, the effect of LPS on cell viability is double-sided. Not only proliferation inhibition from LPS is well known, but also promotion of LPS on cellular proliferation and survival was also reported. It was because that LPS could play a role in the cell pyroptosis, which has a close relationship with acute lung injury, therefore LPS is investigated as a therapy of cancer with its inhibition of cellular proliferation and apoptosis promotion through the caspase-11/NLRP3 inflammasome pathway (Li et al., 2019a). Similarly, Shigetoshi Yokoyama etc,. raised that LPS-triggered acute inflammation decreases the proliferative ability of T cells (Hsiung et al., 2020). On the other hand, a study focused on *helicobacter* pylori-induced human gastric mucosa diseases revealed that LPS could induce more intense cell proliferation in chronic inflection (4 weeks) than that in acute inflammation (1 week) (Massarrat et al., 2016). Overall, LPS, indeed, has the ability to induce the cellular proliferation especially for chronic infection and the cellular inhibition of LPS are easily observed in acute pathological changes. Furthermore, in our study, the CD4+ T cells were analyzed after 18 h 1 μg/mL LPS challenge, and the inhibition of cells proliferation was observed consequently, which agrees with the outcome observed in acute inflammatory models.

FOXO1, a key transcription factor in apoptosis, induces the expression of membrane-associated proteins like the FAS ligand and the TNF-related apoptosis-inducing ligand (TRAIL), which activate the death receptor and subsequently caspase 8, thereby initiating the extrinsic apoptosis pathway. Furthermore, FOXO1 can indirectly inhibit the pro-survival protein Bcl-XL, a member of the BCL-2 family, leading to increased mitochondrial permeability and the activation of the intrinsic apoptosis pathway. Notably, Beclin-1, a protein that promotes autophagy, can interact with BCL-2, thereby inhibiting Beclin-1-mediated autophagy. Additionally, FOXO1 indirectly regulates other proteins of the BCL-2 family, influencing the balance between autophagy and apoptosis (Fu and Tindall, 2008). In addition, studies have reported the regulation of cell apoptosis by the PI3K/AKT/FOXO1 pathway, thereby affecting the pathology of many diseases (Du et al., 2020).

Thus, we are the first study to report the effect of different expression levels of miR-223 on the level of autophagy in CD4^+^ T lymphocytes. Regulation of the autophagy in CD4^+^ T lymphocytes by miR-223 establishes a potential target for treating sepsis. However, our study suffers from a few limitations, including the fact that the results of this experiment are from an *in vitro* system and should be verified *in vivo*. Further, we have only suggested a linear mechanism for the miR-223-mediated regulation of CD4^+^ T lymphocyte function. Further investigations are required to uncover the nuances of this regulation. Therefore, we demonstrate that miR-223 affects the level of autophagy in CD4^+^ T lymphocytes via the regulation of FOXO1, thereby affecting the development of sepsis.

## 5 Conclusion

This study proves that miR-223 participate in the regulation of LPS-induced autophagy via the regulation of FOXO1 expression in CD4^+^ T lymphocytes which shed a new light for the diagnosis and treatment of sepsis.

## Data Availability

The original contributions presented in the study are included in the article/Supplementary Material, further inquiries can be directed to the corresponding authors.

## References

[B1] Bermejo-Martin J. F. Andaluz-Ojeda D. Almansa R. Gandia F. Gomez-Herreras J. I. Gomez-Sanchez E. (2016). Defining immunological dysfunction in sepsis: a requisite tool for precision medicine. J. Infect. 72 (5), 525–536. 10.1016/j.jinf.2016.01.010 26850357

[B2] Boomer J. S. Green J. M. Hotchkiss R. S. (2014). The changing immune system in sepsis: is individualized immuno-modulatory therapy the answer?. Virulence 5 (1), 45–56. 10.4161/viru.26516 24067565 PMC3916383

[B3] Dejager L. Pinheiro I. Dejonckheere E. Libert C. (2011). Cecal ligation and puncture: the gold standard model for polymicrobial sepsis?. Trends Microbiol. 19 (4), 198–208. 10.1016/j.tim.2011.01.001 21296575

[B4] Delano M. J. Ward P. A. (2016). Sepsis-induced immune dysfunction: can immune therapies reduce mortality?. J. Clin. Invest 126 (1), 23–31. 10.1172/JCI82224 26727230 PMC4701539

[B5] Doi K. Leelahavanichkul A. Yuen P. S. Star R. A. (2009). Animal models of sepsis and sepsis-induced kidney injury. J. Clin. Invest 119 (10), 2868–2878. 10.1172/JCI39421 19805915 PMC2752080

[B6] Du L. J. Pang B. Tan Y. M. Yang Y. N. Zhang M. Z. Pang Q. (2020). Banxia xiexin decoction ameliorates t-BHP-induced apoptosis in pancreatic beta cells by activating the PI3K/AKT/FOXO1 signaling pathway. J. Diabetes Res. 2020, 3695689. 10.1155/2020/3695689 32377518 PMC7191444

[B7] Fasseu M. Treton X. Guichard C. Pedruzzi E. Cazals-Hatem D. Richard C. (2010). Identification of restricted subsets of mature microRNA abnormally expressed in inactive colonic mucosa of patients with inflammatory bowel disease. PLoS One 5 (10), e13160. 10.1371/journal.pone.0013160 20957151 PMC2950152

[B8] Franks Z. Carlisle M. Rondina M. T. (2015). Current challenges in understanding immune cell functions during septic syndromes. BMC Immunol. 16, 11. 10.1186/s12865-015-0073-4 25887317 PMC4374283

[B9] Fu Z. Tindall D. J. (2008). FOXOs, cancer and regulation of apoptosis. Oncogene 27 (16), 2312–2319. 10.1038/onc.2008.24 18391973 PMC2819403

[B10] Haneklaus M. Gerlic M. O’Neill L. A. Masters S. L. (2013). miR-223: infection, inflammation and cancer. J. Intern Med. 274 (3), 215–226. 10.1111/joim.12099 23772809 PMC7166861

[B11] Hotchkiss R. S. Monneret G. Payen D. (2013a). Immunosuppression in sepsis: a novel understanding of the disorder and a new therapeutic approach. Lancet Infect. Dis. 13 (3), 260–268. 10.1016/S1473-3099(13)70001-X 23427891 PMC3798159

[B12] Hotchkiss R. S. Monneret G. Payen D. (2013b). Sepsis-induced immunosuppression: from cellular dysfunctions to immunotherapy. Nat. Rev. Immunol. 13 (12), 862–874. 10.1038/nri3552 24232462 PMC4077177

[B13] Hotchkiss R. S. Sherwood E. R. (2015). Immunology. Getting sepsis therapy right. Science 347 (6227), 1201–1202. 10.1126/science.aaa8334 25766219 PMC4398343

[B14] Hsiung S. Moro A. Ban Y. Chen X. Savio A. S. Hernandez R. (2020). Acute lipopolysaccharide-induced inflammation lowers IL-2R signaling and the proliferative potential of regulatory T cells. Immunohorizons 4 (12), 809–824. 10.4049/immunohorizons.2000099 33334814

[B15] Johnnidis J. B. Harris M. H. Wheeler R. T. Stehling-Sun S. Lam M. H. Kirak O. (2008). Regulation of progenitor cell proliferation and granulocyte function by microRNA-223. Nature 451 (7182), 1125–1129. 10.1038/nature06607 18278031

[B16] Kaurani L. Besse M. Methfessel I. Methi A. Zhou J. Pradhan R. (2023). Baseline levels of miR-223-3p correlate with the effectiveness of electroconvulsive therapy in patients with major depression. Transl. Psychiatry 13 (1), 294. 10.1038/s41398-023-02582-4 37699900 PMC10497550

[B17] Li C. C. Munitic I. Mittelstadt P. R. Castro E. Ashwell J. D. (2015). Suppression of dendritic cell-derived IL-12 by endogenous glucocorticoids is protective in LPS-induced sepsis. PLoS Biol. 13 (10), e1002269. 10.1371/journal.pbio.1002269 26440998 PMC4595142

[B18] Li J. Li M. Su L. Wang H. Xiao K. Deng J. (2015). Alterations of T helper lymphocyte subpopulations in sepsis, severe sepsis, and septic shock: a prospective observational study. Inflammation 38 (3), 995–1002. 10.1007/s10753-014-0063-3 25403265

[B19] Li Y. Song D. Bo F. Deng M. Tang X. (2019a). Diazepam inhibited lipopolysaccharide (LPS)-induced pyroptotic cell death and alleviated pulmonary fibrosis in mice by specifically activating GABAA receptor α4-subunit. Biomed. Pharmacother. 118, 109239. 10.1016/j.biopha.2019.109239 31351431

[B20] Li Y. Zhou D. Ren Y. Zhang Z. Guo X. Ma M. (2019b). Mir223 restrains autophagy and promotes CNS inflammation by targeting ATG16L1. Autophagy 15 (3), 478–492. 10.1080/15548627.2018.1522467 30208760 PMC6351131

[B21] Lin M. H. Chen M. C. Chen T. H. Chang H. Y. Chou T. C. (2015). Magnolol ameliorates lipopolysaccharide-induced acute lung injury in rats through PPAR-gamma-dependent inhibition of NF-kB activation. Int. Immunopharmacol. 28 (1), 270–278. 10.1016/j.intimp.2015.05.051 26072062

[B22] Luan Y. Y. Yao Y. M. Xiao X. Z. Sheng Z. Y. (2015). Insights into the apoptotic death of immune cells in sepsis. J. Interferon Cytokine Res. 35 (1), 17–22. 10.1089/jir.2014.0069 25007137 PMC4291200

[B23] Massarrat S. Saniee P. Siavoshi F. Mokhtari R. Mansour-Ghanaei F. Khalili-Samani S. (2016). The effect of helicobacter pylori infection, aging, and consumption of proton pump inhibitor on fungal colonization in the stomach of dyspeptic patients. Front. Microbiol. 7, 801. 10.3389/fmicb.2016.00801 27252698 PMC4879133

[B24] Menden H. Tate E. Hogg N. Sampath V. (2013). LPS-mediated endothelial activation in pulmonary endothelial cells: role of Nox2-dependent IKK-β phosphorylation. Am. J. Physiol. Lung Cell Mol. Physiol. 304 (6), L445–L455. 10.1152/ajplung.00261.2012 23333803 PMC3602745

[B25] Ni Y. F. Jiang T. Cheng Q. S. Gu Z. P. Zhu Y. F. Zhang Z. P. (2012). Protective effect of magnolol on lipopolysaccharide-induced acute lung injury in mice. Inflammation 35 (6), 1860–1866. 10.1007/s10753-012-9507-9 23053725

[B26] Seymour C. W. Liu V. X. Iwashyna T. J. Brunkhorst F. M. Rea T. D. Scherag A. (2016). Assessment of clinical criteria for sepsis: for the third international consensus definitions for sepsis and septic shock (Sepsis-3). JAMA 315 (8), 762–774. 10.1001/jama.2016.0288 26903335 PMC5433435

[B27] Shankar-Hari M. Bertolini G. Brunkhorst F. M. Bellomo R. Annane D. Deutschman C. S. (2015). Judging quality of current septic shock definitions and criteria. Crit. Care 19, 445. 10.1186/s13054-015-1164-6 26702879 PMC4699344

[B28] Stevenson E. K. Rubenstein A. R. Radin G. T. Wiener R. S. Walkey A. J. (2014). Two decades of mortality trends among patients with severe sepsis: a comparative meta-analysis. Crit. Care Med. 42 (3), 625–631. 10.1097/CCM.0000000000000026 24201173 PMC4313930

[B29] Stieglitz D. Schmid T. Chhabra N. F. Echtenacher B. Mannel D. N. Mostbock S. (2015). TNF and regulatory T cells are critical for sepsis-induced suppression of T cells. Immun. Inflamm. Dis. 3 (4), 374–385. 10.1002/iid3.75 26734459 PMC4693718

[B30] Walkey A. J. Lagu T. Lindenauer P. K. (2015). Trends in sepsis and infection sources in the United States. A population-based study. Ann. Am. Thorac. Soc. 12 (2), 216–220. 10.1513/AnnalsATS.201411-498BC 25569845 PMC4342831

[B31] Wang H. Li X. Zhang Q. Fu C. Jiang W. Xue J. (2023). Autophagy in disease onset and progression. Aging Dis. 15, 1646–1671. 10.14336/AD.2023.0815 PMC11272186 37962467

[B32] Wang H. Zhang P. Chen W. Feng D. Jia Y. Xie L. (2012). Serum microRNA signatures identified by Solexa sequencing predict sepsis patients' mortality: a prospective observational study. PLoS One 7 (6), e38885. 10.1371/journal.pone.0038885 22719975 PMC3376145

[B33] Wang H. J. Wang B. Z. Zhang P. J. Deng J. Zhao Z. R. Zhang X. (2014). Identification of four novel serum protein biomarkers in sepsis patients encoded by target genes of sepsis-related miRNAs. Clin. Sci. (Lond) 126 (12), 857–867. 10.1042/CS20130301 24303815 PMC4202716

[B34] Wang J. F. Yu M. L. Yu G. Bian J. J. Deng X. M. Wan X. J. (2010). Serum miR-146a and miR-223 as potential new biomarkers for sepsis. Biochem. Biophys. Res. Commun. 394 (1), 184–188. 10.1016/j.bbrc.2010.02.145 20188071

[B35] Yoshii S. R. Mizushima N. (2017). Monitoring and measuring autophagy. Int. J. Mol. Sci. 18 (9), 1865. 10.3390/ijms18091865 28846632 PMC5618514

