# Corrigendum: FOXO1-mediated autophagy regulation by miR-223 in sepsis-induced immunosuppression

**DOI:** 10.3389/fphar.2024.1533534

**Published:** 2024-12-06

**Authors:** Guoan Xiang, Qi Li, Di Lian, Chengcheng Su, Xin Li, Shoulong Deng, Lixin Xie

**Affiliations:** ^1^ College of Pulmonary and Critical Care Medicine, Chinese PLA General Hospital, Beijing, China; ^2^ Chinese PLA Medical School, Beijing, China; ^3^ Department of Tuberculosis, Beijing Chest Hospital, Capital Medical University, Beijing, China; ^4^ Department of Respiratory and Critical Care Medicine, Pingjin Hospital, Characteristic Medical Center of the Chinese People’s Armed Police Force, Tianjin, China; ^5^ Department of Emergency, Third Medical Center of Chinese PLA General Hospital, Beijing, China; ^6^ National Center of Technology Innovation for Animal Model, National Human Diseases Animal Model Resource Center, National Health Commission of China (NHC) Key Laboratory of Comparative Medicine, Institute of Laboratory Animal Sciences, Chinese Academy of Medical Sciences and Comparative Medicine Center, Peking Union Medical College, Beijing, China

**Keywords:** sepsis, miR-223, CD4^+^ T lymphocytes, autophagy, FOXO1, immunosuppression

In the published article, there was an error in the **Funding** statement. “The Natural Science Foundation of Shandong Province (ZR2021MH388)” was erroneously included. The correct **Funding** statement appears below.

