# Supplementary figures and images for: FOXO1-mediated autophagy regulation by miR-223 in sepsis-induced immunosuppression
Source: Front Pharmacol. 2024 Oct 8;15:1469286. doi: 10.3389/fphar.2024.1469286 (PMC11493625; doi:10.3389/fphar.2024.1469286)

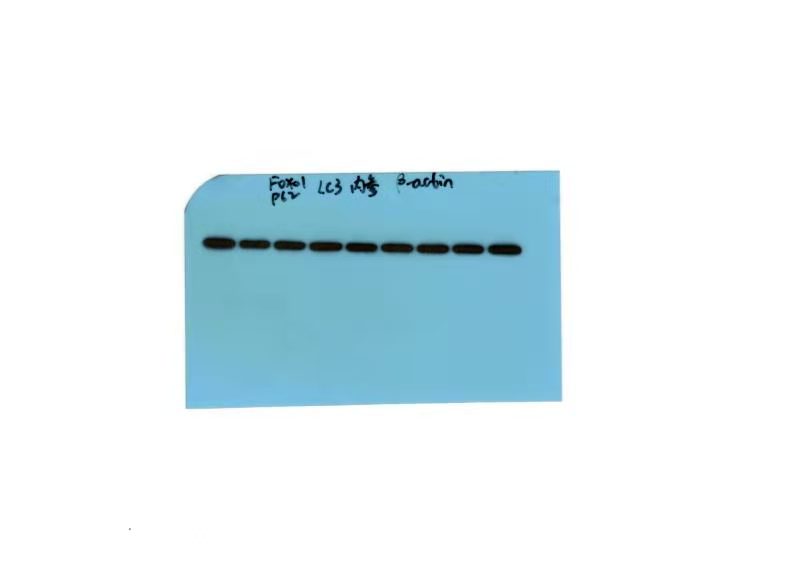

Supplement: Supplementary file 2 [file DataSheet2.ZIP › Original Images for Blots-fig7/fig7-Original Images-╬▓-actin.jpg]

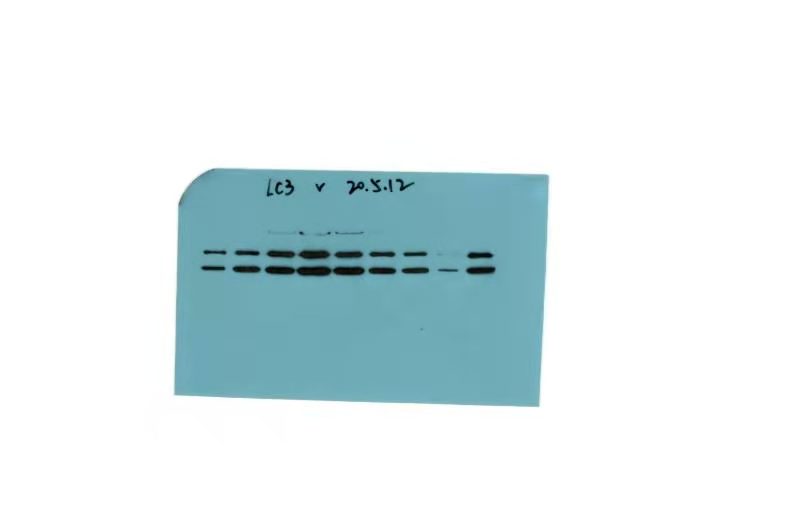

Supplement: Supplementary file 2 [file DataSheet2.ZIP › Original Images for Blots-fig7/fig7-Original Images-LC3B.jpg]

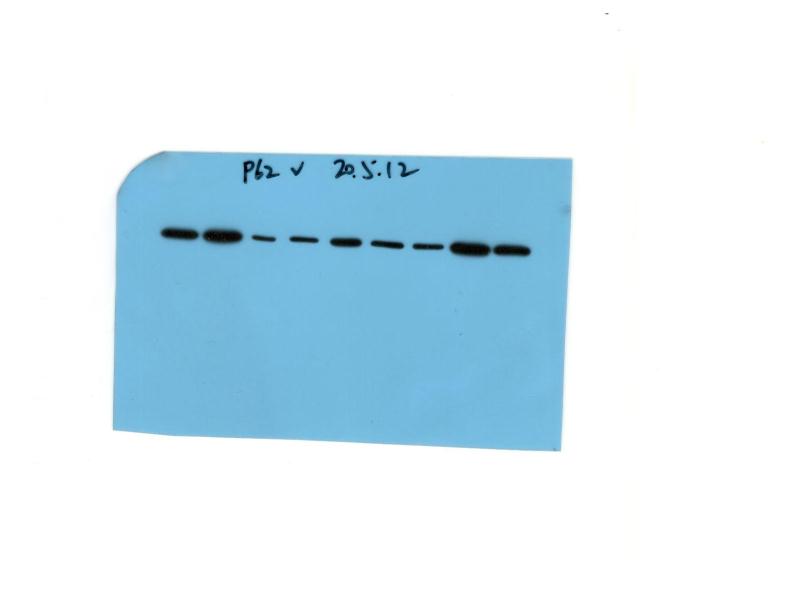

Supplement: Supplementary file 2 [file DataSheet2.ZIP › Original Images for Blots-fig7/fig7-Original Images-p62.jpg]

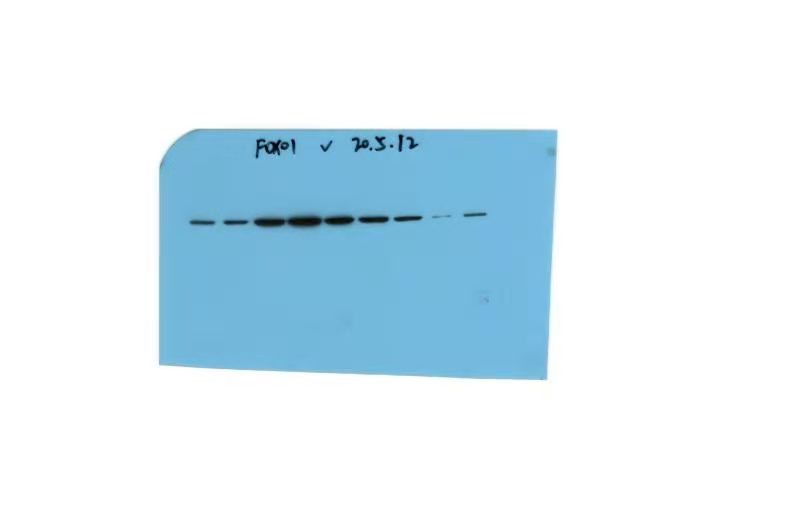

Supplement: Supplementary file 2 [file DataSheet2.ZIP › Original Images for Blots-fig7/fig7-Original Images-FOXO1.jpg]

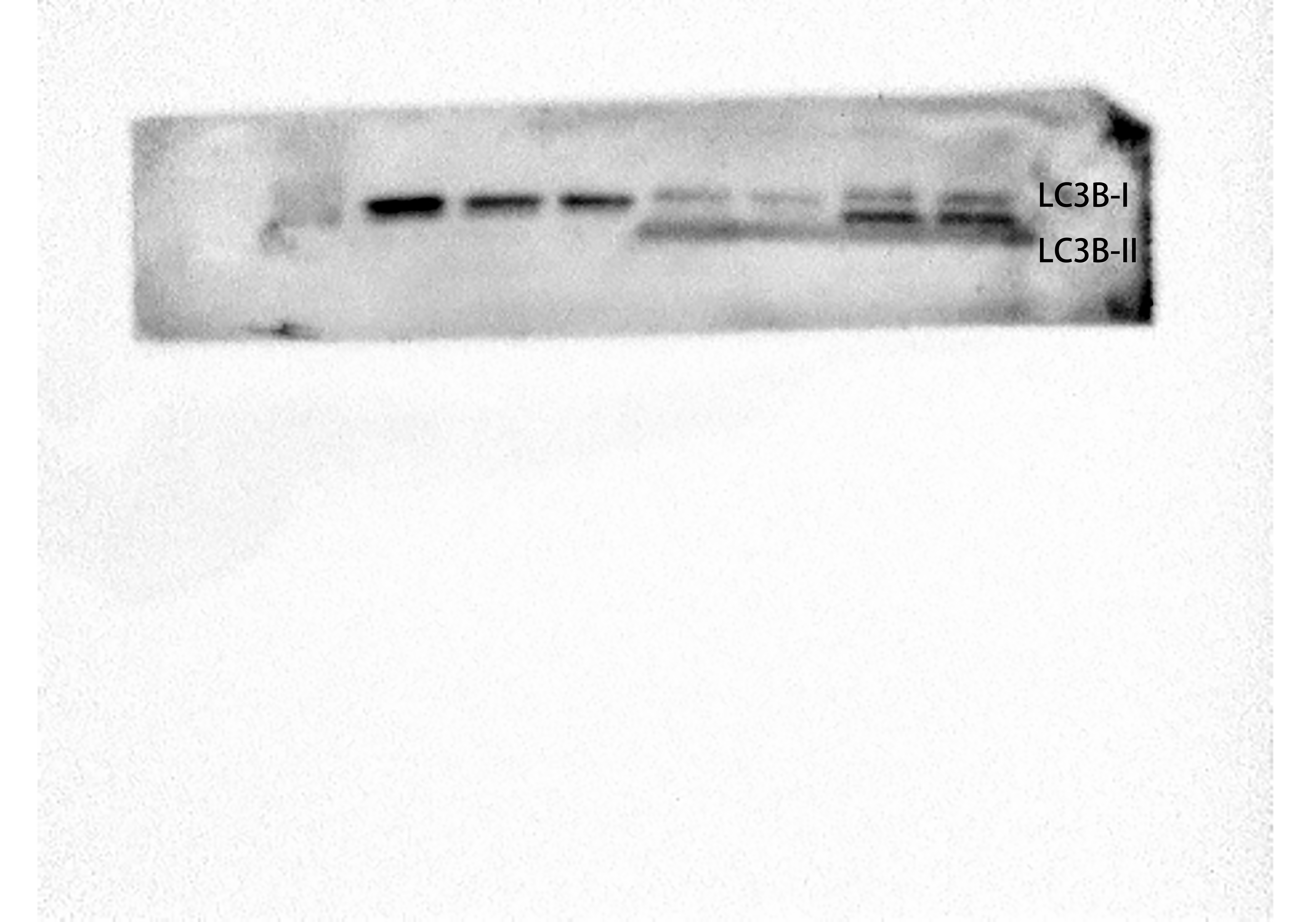

Supplement: Supplementary file 2 [file DataSheet2.ZIP › Original Images for Blots-fig8/fig8-Original Images-LC3B.jpg]

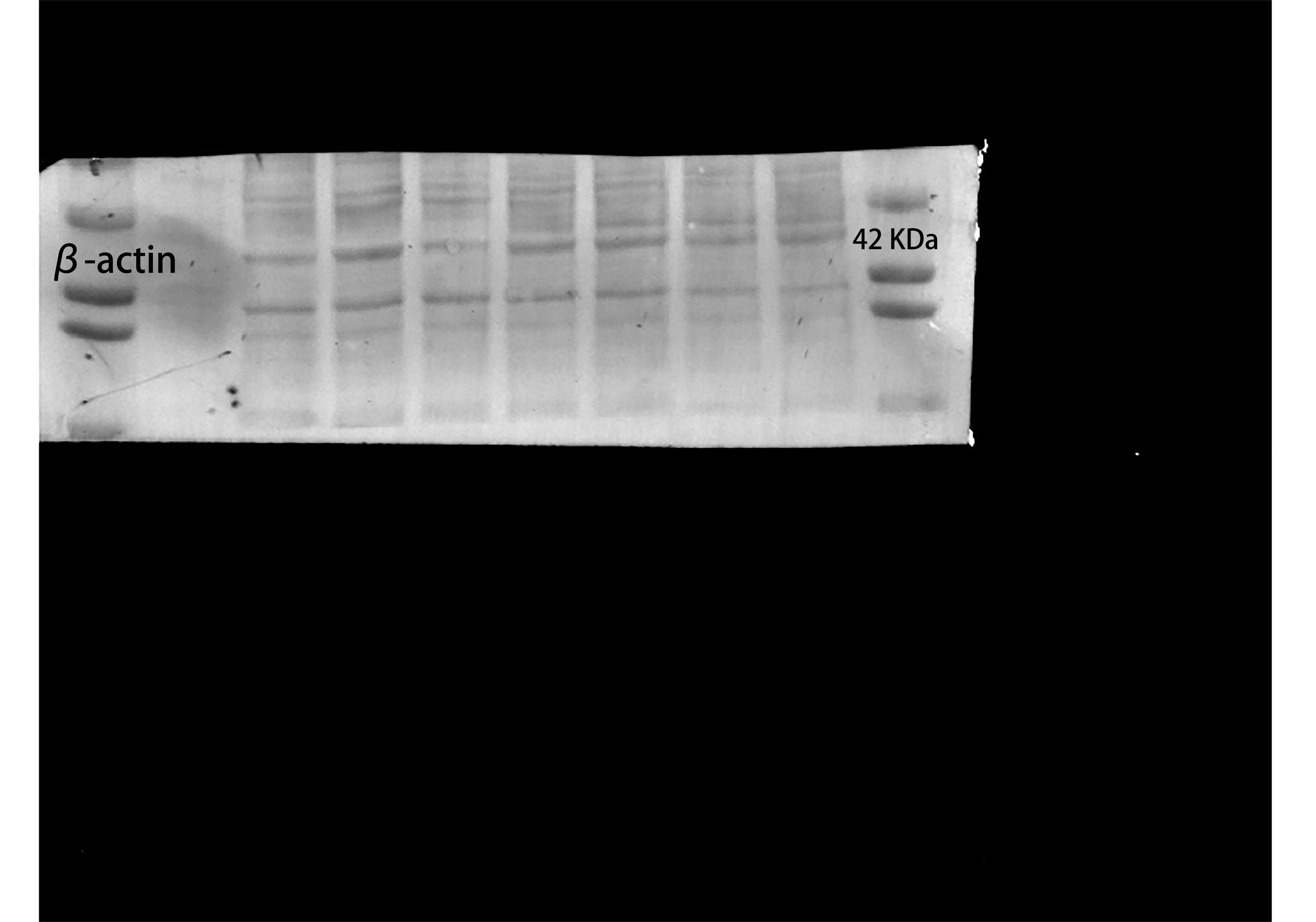

Supplement: Supplementary file 2 [file DataSheet2.ZIP › Original Images for Blots-fig8/fig8-Original Images-actin.jpg]

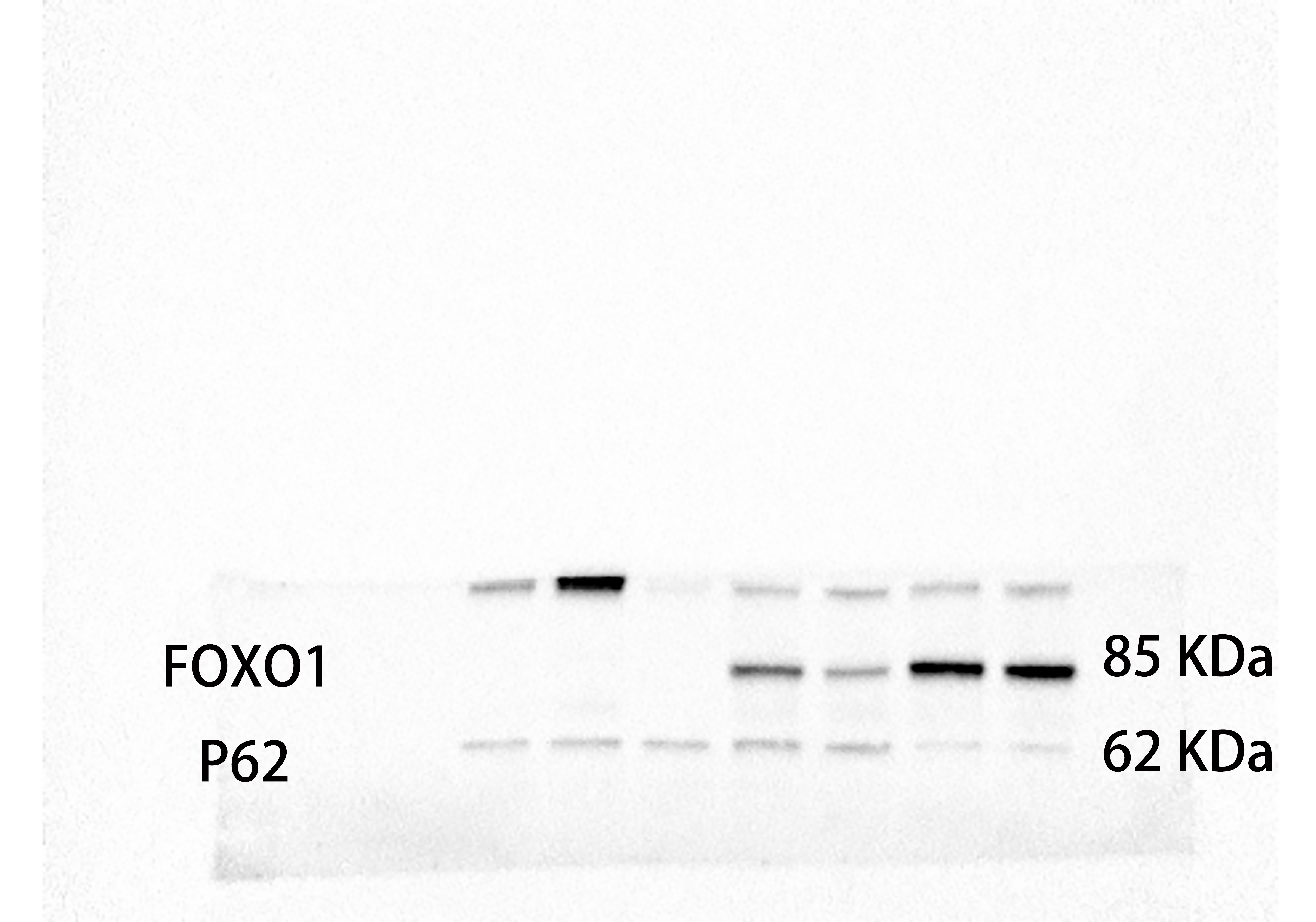

Supplement: Supplementary file 2 [file DataSheet2.ZIP › Original Images for Blots-fig8/fig8-Original Images-FOXO1-P62.jpg]
